# Metastatic Intraocular Tumor Due to Colorectal Adenocarcinoma: Case Report and Literature Review

**DOI:** 10.18502/jovr.v15i4.7794

**Published:** 2020-10-25

**Authors:** Deivy Cruzado-Sanchez, Luis A. Saavedra-Mejia, Walter A. Tellez, Grissnery Maquera-Torres, Solon Serpa-Frias

**Affiliations:** ^1^Ophthalmic Oncology Service, Instituto Nacional de Enfermedades Neoplásicas, Lima, Peru; ^2^Sociedad Científica de Estudiantes de Medicina Villarrealinos, Universidad Nacional Federico Villarreal, Lima, Peru; ^3^Pathology Service, Instituto Nacional de Enfermedades Neoplásicas, Lima, Peru

**Keywords:** Colorectal Neoplasms, Eye Neoplasms, Neoplasm Metastasis

## Abstract

**Purpose:**

To describe the clinical and histopathological findings of a case of intraocular metastasis due to colorectal adenocarcinoma and to carry out a literature review.

**Case Report:**

A 64-year-old man with a history of tumor resection due to infiltrating colorectal adenocarcinoma three years previously sought ophthalmological care because of severe ocular pain without response to medical treatment and progressive vision loss in the left eye. On ultrasonographic examination, there was a heterogeneous intraocular choroidal tumor, which occupied approximately 40% of the vitreous cavity, as well as peritumoral serous retinal detachment. The patient underwent left eyeball enucleation. The histopathological diagnosis was metastatic tubular adenocarcinoma involving the retina and choroid that partially infiltrated the sclera and the proximal optic nerve.

**Conclusion:**

The present case highlights a rare pathological entity associated with variable therapeutic schemes and survival times and poor prognosis in patients with metastatic intraocular tumors due to colorectal adenocarcinoma.

##  INTRODUCTION

Colon cancer is the second cause of death associated with cancer in developed countries, representing 9% of all cancer estimated deaths;^[1]^ while in developing countries like Peru, it is the fifth cause of death, accounting for 6 deaths per 100,000 inhabitants.^[2]^ The most frequent sites of metastasis are liver (77%), peritoneum (25%), and lungs (22%), while intraocular involvement is infrequent and rarely reported.^[3,4,5]^

The aim of this report is to describe the clinical and histopathological findings of a case of intraocular metastasis from colorectal adenocarcinoma and to carry out a literature review.

##  CASE REPORT

**Figure 1:**
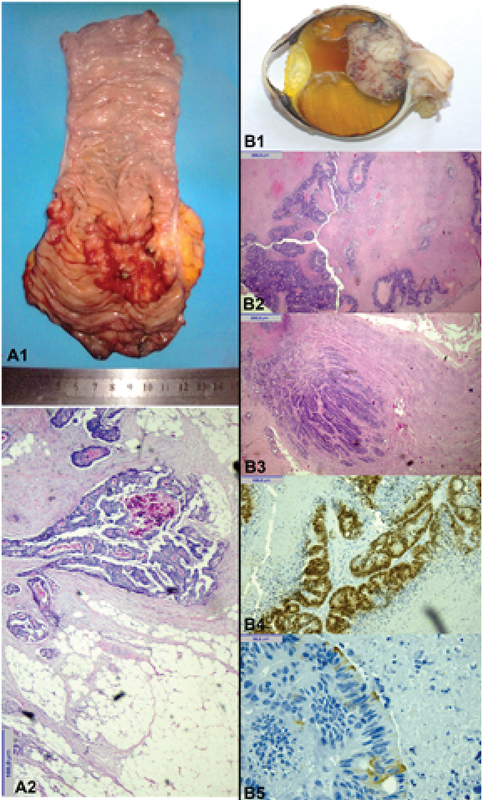
Primary neoplasms from A1 to A3. (A1) Colonic macroscopy of a single tumor above the junction of the rectum and sigmoid. (A2) Tubular adenocarcinoma moderately differentiated in the submucosal layer of colon pT3N1b. Intraocular metastasis from B1 to B5. (B1) Eyeball with exophytic tumor. H&E staining. (B2) Metastasis of tubular adenocarcinoma in the retina. (B3) Metastasis of tubular adenocarcinoma in the optic nerve. (B4) Positive nuclear staining for CDX2. (B5) Positive focal expression of CK20.

The patient was a 64-year-old man with six months of severe and progressive vision loss in the left eye associated with severe ocular pain. He had a history of resection of a moderately differentiated colorectal adenocarcinoma with muscle layer involvement, invasive borders, and involvement in 3 of the 25 regional lymph nodes (T3N1M0) three years ago. He refused complementary treatment with chemotherapy at that time (Figures 1A1 and 1A2).

**Figure 2:**
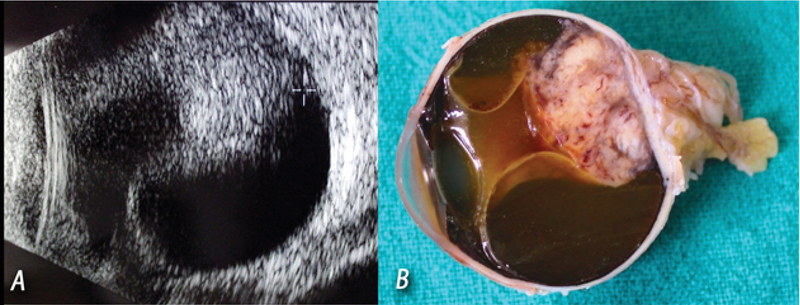
(A) Ocular ultrasound demonstrating a heterogeneous intraocular choroidal tumor associated with retinal detachment. (B) Enucleated surgical tissue from the eye with a whitish tumor mass with signs of hemorrhage.

The ophthalmological examination showed a visual acuity (VA) of 20/20 in the right eye and no light perception in the left eye. Intraocular pressure was 16 mmHg in the right eye and 50 mmHg in the left eye. Biomicroscopic examination showed moderate conjunctival hyperemia, mydriatic and unreactive pupil, moderate corneal edema, and moderate crystalline opacity in the left eye and unremarkable findings in the right eye. Funduscopy revealed extensive whitish tumor mass with multiple hemorrhagic foci on its surface, moderate vitreous opacity and retinal detachment in the left eye. On ultrasonographic examination, there was a heterogeneous intraocular choroidal tumor, which occupied approximately 40% of the vitreous cavity, as well as peritumoral serous retinal detachment (Figure 2). Computed tomography preformed for metastasis work-up demonstrated tumoral lesions in the lungs.

He was diagnosed as a secondary non-controlled glaucoma due to probable metastatic choroidal tumor. He received maximum antihypertensive ocular treatment in the left eye with poor response and persistence of pain. Due to the evidence of a blind, painful eye with a large intraocular tumor, left eyeball enucleation was performed with patient's informed consent. The histopathological diagnosis was metastatic tubular adenocarcinoma involving the retina and choroid, partially infiltrating the sclera and the proximal optic nerve. Immunohistochemical study was positive to CDX2; some tumor cells expressed CK20 focally, and the tumor was negative for CK7. All these findings were consistent with a primary colorectal adenocarcinoma as the source of metastasis (Figures 1B1–1B5).

Medical oncology team assessed the patient. Treatment with chemotherapy and palliative radiotherapy was indicated, but he refused it and just accepted palliative pain therapy. He died six months after the enucleation.

##  DISCUSSION

Metastasis of colorectal carcinoma to the eye is infrequent,^[3]^ and it is associated with advanced stages of the disease with an unfavorable prognosis and poor survival.^[6]^

We performed a literature review through a systematic search in PubMed and Google Scholar using an appropriate search strategy for each database (supplementary material 1) and reviewed the references of the reports included in the systematic search to increase the chances of identifying all reported cases of intraocular metastasis due to colorectal adenocarcinoma. We included 23 case reports and 1 case series (Table 1), accounting for 25 cases, for which 19 patients presented with metastasis only to the choroid,^[4,5,6,7,8,9,10,11,12,13,14,15,16,17,18,19,20,21,22,23]^ three with metastasis only to the retina,^[24,25,26]^ one with metastasis to the retina and choroid,^[27]^ one with metastasis to the sclera, retina, and optic nerve,^[28]^ and one with metastasis to the choroid and optic nerve.^[29]^ The average age was 55.2 years (ranging from 30 to 80 years), and 15 patients were men. Regarding the source of the primary neoplasia, 12 were derived from the colon, 12 from the rectum, and 1 was colorectal. However, most of the papers did not report the TNM staging.

**Table 1:** Characteristics of previous intraocular metastasis cases due to colorectal adenocarcinoma reported in the literature


**Author**	**Year of publication**	**Sex**	**Age**	**Colorectal cancer**		**Intraocular metastasis**				**Other sites of metastasis**	**Survival time (months)**
		**Primary lesion**	**TNM or stage**	**Intraocular site**	**Time to diagnosis (months)**	**Symptom**	**Treatment**	
Kennedy^[24]^	1958	M	51	Rectosigmoid	NS	Retina	S	Blurred vision	Enucleation	None	9
Howard^[27]^ ${}^{*}$	1968	M	63	Colon	NS	Retina, choroid	36	Visual disturbance	Enucleation	Orbit	16
Cole^[7]^ ${}^{*}$	1985	F	48	Rectum	T4a	Choroid	23	Blurred vision	RT, CT	Lung	4
Tano^[8]^ ${}^{*}$	1989	M	30	Rectum	NS	Choroid	S	Blurred vision	Enucleation	Bone, skin	4
Endo^[9]^ ${}^{*}$	1997	F	49	Rectum	NS	Choroid	84	Flashes	Enucleation	Liver, lung	3
Ward^[10]^	2000	F	52	Colon	NS	Choroid	S	Vision loss	None	Intraabdominal	1
Nakamura^[11]^	2002	M	79	Colon	IIIb	Choroid	18	Blurred vision	CT	Lung	NS
Fujiwara^[12]^ ${}^{*}$	2004	M	53	Rectum	NS	Choroid	30	Vision loss	RT, CT	Liver, lung, bone	1
Linares^[13]^	2004	M	47	Rectum	NS	Choroid	S	Blurred vision	RT, CT	Liver, lung	9
Apte^[25]^	2005	M	39	Colon	NS	Retina	3	Visual disturbance	RT, CT	Liver, lung	7
Hisham^[28]^	2006	F	32	Rectum	NS	Sclera, retina, optic nerve	10	Ocular pain	RT	Spine, breast, orbit	0.5
Kuo^[14]^	2008	F	65	Colon	NS	Choroid	20	Vision loss	Intravitreous Bev	Brain	5
Sashiyama^[15]^	2010	M	49	Rectum	NS	Choroid	15	Vision loss	CT	Lung, bone	11
Lin^[16]^	2010	M	43	Colon	NS	Choroid	96	Vision loss	CT, intravitreous Bev	Bone	4
Neale^[17]^	2010	M	43	Rectum	T2N0M0	Choroid	18	Blurred vision	NS	Lung, pelvis, brain	NS
Miyake^[18]^ ${}^{*}$	2012	M	74	Rectum	NS	Choroid	S	Vision loss	CT	Liver, lung	8
Tei^[19]^	2014	M	60	Rectum	T1	Choroid	30	Floaters	RT	Lung	27
Maudgil^[20]^	2015	F	57	Colon	NS	Choroid	18	Vision loss	Intravitreous Bev	NS	NS
	2015	M	80	Colon	NS	Choroid	6	Vision loss	Intravitreous Bev	Liver	NS
Kawhaja^[4]^	2015	F	60	Rectum	T3N1M0	Choroid	42	Flashes	RT, CT, systemic Bev	Lung	31
Huo^[21]^	2015	F	51	Colon	T3N1M0	Choroid	27	Ocular redness, foreign body sensation	RT	Lung, bone	2.5
Nookala^[26]^	2016	M	56	Colon	T3N0Mx	Retina	25	Vision loss and ocular pression sensation	RT	Liver, lung	NS
Boss^[22]^	2016	F	68	Rectum	T3N0Mx	Choroid	96	Floaters and flashes	intravitreous Bev	Lung, cerebellum	NS
Ha^[23]^	2016	F	78	Colon	T3N0M0	Choroid	6	Visual disturbance	CT	Lung, skin	8
Walker^[29]^	2017	M	54	Colon	T1N0M0	Choroid, optic nerve	16	Vision loss	RT, systemic Bev, CT, photodynamic therapy	Lung	24
Present case	2018	M	64	Rectosigmoid	T3N1M0	Choroid, retina, optic nerve, sclera	36	Ocular pain and vision loss	Enucleation	Lung	6
**PubMed**	("neoplasm metastasis"[MeSH Terms] OR "metastasis"[Title/Abstract] OR "metastases"[Title/Abstract] OR "ocular metastasis"[Title/Abstract] OR "intraocular metastasis"[Title/Abstract]) AND ("choroid"[MeSH Terms] OR "choroid"[Title/Abstract] OR "retina"[MeSH Terms] OR "retina"[Title/Abstract]) AND ("case reports"[Publication Type] OR "case study"[Title/Abstract] OR "case report"[Title/Abstract])	October 2018	593
**Google Scholar**	("neoplasm metastasis" OR "metastasis" OR "metastases" OR "ocular metastasis" OR "intraocular metastasis") AND ("choroid" OR "retina") AND (“colorectal cancer” OR “colon cancer” OR “colonic neoplasm” OR “colonic cancer”)	October 2018	17200
*The data from these articles were extracted secondarily from other articles that cited them because it was not possible to access to the full-text article. M, male; F, female; NS, not specified; RT, radiotherapy; CT, chemotherapy; Bev, bevacizumab; S, simultaneous			

The average time of detection of intraocular metastasis after the diagnosis of the primary colorectal neoplasm was 24.7 months (ranging from immediately up to 96 months); only in five cases was the diagnosis of intraocular metastasis made at the same time as the primary colorectal neoplasm diagnosis.^[8,10,13,18,24]^ In addition to intraocular metastasis, an involvement of other organs have also been found, such as liver, lung, skin, bone, brain, and cerebellum (Table 1). In the current case, intraocular metastasis was detected 36 months after the diagnosis of the primary tumor.

In the cases reported in the literature (Table 1), the most frequent reason for ophthalmological consultation was some type of vision dysfunction (decreased VA, blurred vision, floaters or flashes), whereas in the present case, the patient had severe ocular pain related to uncontrolled secondary glaucoma in addition to vision loss, similar to a case reported in Malaysia.^[28]^

Four cases of eyeball enucleation have been reported in the literature.^[8,9,24,27]^ Currently, enucleation is considered a reserved therapeutic option for intraocular malignant tumors in advanced stages with extensive ocular involvement and severe pain due to secondary glaucoma.^[30,31]^ In the present case, enucleation was performed because of the extensive ocular involvement, absent visual function, secondary uncontrollable glaucoma, and the refusal of the patient to submit to other therapeutic proposals.

The survival time ranged from 14 days to 31 months after the diagnosis of intraocular metastasis in previously published reports (Table 1). In the present case, the patient's survival time was six months. In previous cases treated with enucleation,^[8,9,24,27]^ the survival time ranged from 3 to 16 months, while longer survival times were reported in patients treated with radiotherapy, chemotherapy, and systemic bevacizumab treatment (24 and 31 months).

In the present case, the immunohistochemical assessment of the intraocular tumor was positive for CDX2, focally positive for CK20, and negative for CK7. These results showed an immunohistochemical profile with high sensitivity and specificity for colorectal adenocarcinoma (CDX2+, CK7-/CK20+).^[32]^ The focal positivity of the CK20 marker is consistent with the pattern of expression in colorectal adenocarcinoma, with greater expression in rectal carcinomas than in nonrectal carcinomas,^[32]^ so the focal expression in this case may be due to the greater expression of this marker in tumor cells derived from the rectum than in those derived from the sigmoid colon.

In conclusion, the present case highlights a rare pathological entity that has been increasingly reported in recent years and has been observed in relation to variable therapeutic schemes and survival times, and simultaneous metastasis to other organs has also been observed. Therefore, clinicians should consider the possibility of intraocular metastasis when managing patients with colorectal cancer in advanced stages.

## References

[1] Siegel R, Miller K, Jemal A. Cancer statistics, 2017. *CA Cancer J Clin* 2017;67:7–30. 10.3322/caac.21387 28055103

[2] Dirección General de Epidemiología. Análisis de la situación del cáncer en el Perú 2013. Ministerio de Salud [Internet]. Lima: Ashka; 2013 [cited August 28, 2017]. Available from: http://www.dge.gob.pe/portal/docs/asis_cancer.pdf

[3] Hess KR, Varadhachary GR, Taylor SH, Wei W, Raber MN, Lenzi R, et al. Metastatic patterns in adenocarcinoma. *Cancer* 2006;106:1624–1633. 10.1002/cncr.21778 16518827

[4] Khawaja M, Minturn J, Spittler A, Chiorean E. Ocular metastasis of colorectal cancer: an uncommon presentation of a common malignancy. *Hematol Oncol Stem Cell Ther* 2015;8:176–180. 10.1016/j.hemonc.2015.02.002 25784129

[5] Shields CL, Shields JA, Gross NE, Schwartz GP, Lally SE. Survey of 520 eyes with uveal metastases. *Ophthalmology* 1997;104:1265–1276. 10.1016/s0161-6420(97)30148-1 9261313

[6] Konstantinidis L, Damato B. Intraocular metastases – a review. *Asia Pac J Ophthalmol* 2017;6:208–214. 10.22608/APO.201712 28399345

[7] Cole MD, Farah NB. The choroid – an unusual site for metastasis in patients with adenocarcinoma of the rectum – a case report. *Eur J Surg Oncol* 1985;11:275–278. 4029408

[8] Tano S, Hayashi H, Momoeda S. Metastasis of rectal carcinoma to the choroid: a case report. *Nihon Ganka Kiyo* 1989;40:1284–1288.

[9] Endo H, Tajika T, Takebayashi H, Shiota H, Yoshida M, Kudo E. A case report of choroidal metastasis from rectal cancer. *Ganka Rinsho Iho* 1997;91:1141.

[10] Ward SD, Byrne BJ, Kincaid MC, Mann ES. Ultrasonographic evidence of a mushroom-shaped choroidal metastasis. *Am J Ophthalmol* 2000;130:681–682. 10.1016/s0002-9394(00)00604-8 11078856

[11] Nakamura H, Harada A, Sakakibara T, Ishikawa T, Yaguchi T, Murakami Y. Metastatic choroidal tumor from cancer of the ascending colon - a case report. *J Jpn Surg Assoc* 2002;63:1031–1035.

[12] Fujiwara T, Machida S, Murai K, Tazawa Y, Baba Y, Shimooki O. A case of choroidal tumor metastasized from rectal cancer. *Ganka* 2004;46:1099–1103.

[13] Linares P, Castanon C, Vivas S, Diz P, Garcia-Palomo A, Llano C, et al. Bilateral choroidal metastasis as the initial manifestation of a rectal cancer. *J Gastroenterol Hepatol* 2004;19:726–727. 10.1111/j.1440-1746.2004.03465.x 15151641

[14] Kuo IC, Haller JA, Maffrand R, Sambuelli RH, Reviglio VE. Regression of a subfoveal choroidal metastasis of colorectal carcinoma after intravitreous bevacizumab treatment. *Arch Ophthalmol* 2008;126:1311–1313. 10.1001/archophthalmol.2008.2 18779499

[15] Sashiyama H, Abe Y, Sasagawa S, Hanada H, Hatori Y, Kubota M, et al. A case of choroidal metastasis from rectal cancer manifesting visual loss as the initial recurrence symptom. *Jpn J Gastroenterol Surg* 2010;43:746–751.

[16] Lin CJ, Li KH, Hwang JF, Chen SN. The effect of intravitreal bevacizumab treatment on choroidal metastasis of colon adenocarcinoma – case report. *Eye* 2010;24:1102–1103. 10.1038/eye.2009.257 19893587

[17] Neale JA, Valsdottir E, Zeger E, Shields C, Marks J. Cerebral and choroidal metastases with retinal detachment, secondary to rectal cancer: a case report. *World J Colorectal Surg* 2010;2:1–9.

[18] Miyake E, Moriwaki M, Sunada T, Takemura J. Regression of choroidal metastasis from rectal cancer following chemotherapy. *Atarashii Ganka* 2012;29:701–704.

[19] Tei M, Wakasugi M, Akamatsu H. Choroidal metastasis from early rectal cancer: case report and literature review. *Int J Surg Case Rep* 2014;5:1278–1281. 10.1016/j.ijscr.2014.10.059 PMC4276086 25460493

[20] Maudgil A, Sears KS, Rundle PA, Rennie IG, Salvi SM. Failure of intravitreal bevacizumab in the treatment of choroidal metastasis. *Eye* 2015;29:707–711. 10.1038/eye.2015.21 PMC4429274 25771814

[21] Huo SM, An HJ, Lee JE, Eum S, Kim MY, Jang YN, et al. Choroidal metastasis from colon cancer treated with palliative radiotherapy. *Korean J Med* 2015;89:723–727.

[22] Boss JD, Lieu P, Tewari A. Effect of treatment of rectal cancer metastasis with intravitreal bevacizumab (Avastin) in a patient with subretinal fluid and macular oedema: short-term follow-up. *BMJ Case Rep* 2016;2016. doi: 10.1136/bcr-2016-216273 10.1136/bcr-2016-216273 PMC5020712 27591037

[23] Ha JY, Oh EH, Jung MK, Park SE, Kim JT, Hwang IG. Choroidal and skin metastases from colorectal cancer. *World J Gastroenterol* 2016;22:9650–9653. 10.3748/wjg.v22.i43.9650 PMC5116609 27920486

[24] Kennedy RJ, Rummel WD, McCarthy JL, Hazard JB. Metastatic carcinoma of the retina; report of a case and the pathologic findings. *AMA Arch Ophthalmol* 1958;60:12–18. 10.1001/archopht.1958.00940080026003 13544659

[25] Apte RS, Dibernardo C, Pearlman JR, Patel S, Schachat AP, Green WR, et al. Retinal metastasis presenting as a retinal hemorrhage in a patient with adenocarcinoma of the cecum. *Arch Ophthalmol* 2005;123:850–853. 10.1001/archopht.123.6.850 15955988

[26] Nookala R, Batchu VV, Lee HM, Loghmani A, Chhabra GS. Difficult diagnosis of colon adenocarcinoma metastasis to retina: a case report and literature review. *Int J Hematol Oncol Stem Cell Res* 2016;10:186–190. PMC4969563 27489594

[27] Howard GM. Retinal hole in an eye with choroidal metastasis. *Trans Am Acad Ophthalmol Otolaryngol *1968;72:186–190. 5659899

[28] Hisham RB, Thuaibah H, Gul YA. Mucinous adenocarcinoma of the rectum with breast and ocular metastases. *Asian J Surg* 2006;29:95–97. 10.1016/S1015-9584(09)60115-9 16644510

[29] Walker CR, Reichstein DA. Bilateral metastases of colorectal cancer to the choroid and optic disc. *Int J Ophthalmol Vis Sci* 2017;2:65–68.

[30] Koylu MT, Gokce G, Uysal Y, Ceylan ML, Akıncıoglu D, Gunal A. Indications for eye removal surgeries. A 15-year experience at a tertiary military hospital. *Saudi Med J* 2015;36:2015–2019. 10.15537/smj.2015.10.12031 PMC4621727 26446332

[31] Kanthan GL, Jayamohan J, Yip D, Conway RM. Management of metastatic carcinoma of the uveal tract: an evidence-based analysis. *Clin Exp Ophthalmol* 2007;35:553–565. 10.1111/j.1442-9071.2007.01550.x 17760639

[32] Bayrak R, Haltas H, Yenidunya S. The value of CDX2 and cytokeratins 7 and 20 expression in differentiating colorectal adenocarcinomas from extraintestinal gastrointestinal adenocarcinomas: cytokeratin 7-/20+ phenotype is more specific than CDX2 antibody. *Diagn Pathol* 2012;7:9. 10.1186/1746-1596-7-9 PMC3331835 22268990

